# [(*Z*)-*O*-Ethyl-*N*-(*o*-tol­yl)thio­carbamato-κ*S*](triphenyl­phosphine-κ*P*)gold(I)

**DOI:** 10.1107/S1600536809048466

**Published:** 2009-11-21

**Authors:** Primjira P. Tadbuppa, Edward R. T. Tiekink

**Affiliations:** a Department of Chemistry, National University of Singapore, Singapore 117543; b Department of Chemistry, University of Malaya, 50603 Kuala Lumpur, Malaysia

## Abstract

The title compound, [Au(C_10_H_12_NOS)(C_18_H_15_P)], features a linear geometry for the Au atom defined by the S and P donor atoms. A small deviation from the ideal geometry is noted and is ascribed to an intra­molecular Au⋯O contact [2.936 (4) Å]. Inversion dimers are formed in the crystal structure mediated by C—H⋯π inter­actions between centrosymmetrically related *o*-tolyl residues [C⋯*Cg* = 3.532 (6) Å].

## Related literature

For structural systematics and luminescence properties of phosphinegold(I) carbonimidothio­ates, see: Ho *et al.* (2006 ▶); Ho & Tiekink (2007 ▶); Kuan *et al.* (2008 ▶). For the synthesis, see Hall *et al.* (1993 ▶).
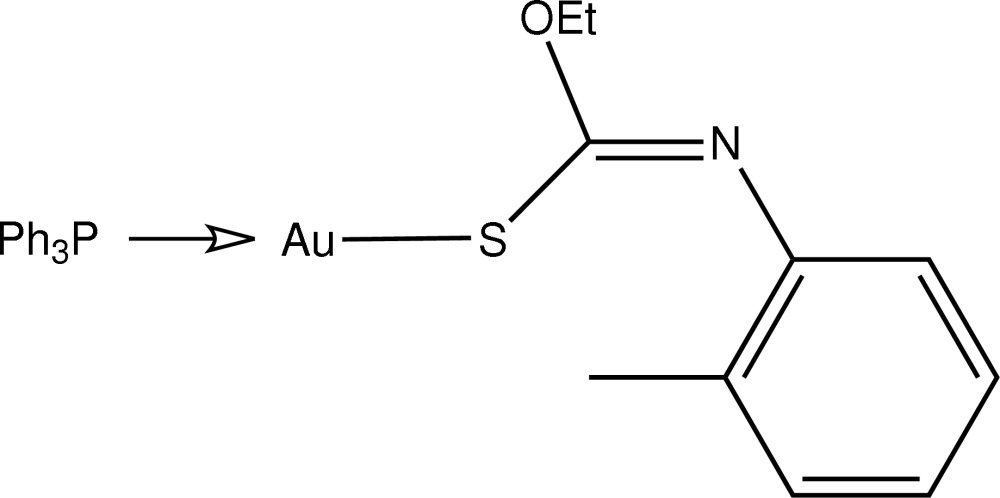


## Experimental

### 

#### Crystal data

[Au(C_10_H_12_NOS)(C_18_H_15_P)]
*M* *_r_* = 653.50
Triclinic, 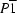

*a* = 9.3378 (6) Å
*b* = 10.1665 (6) Å
*c* = 13.9711 (8) Å
α = 95.514 (1)°
β = 103.371 (1)°
γ = 98.334 (1)°
*V* = 1265.11 (13) Å^3^
*Z* = 2
Mo *K*α radiation
μ = 5.98 mm^−1^
*T* = 223 K
0.24 × 0.13 × 0.03 mm

#### Data collection

Bruker SMART CCD diffractometer
Absorption correction: multi-scan (*SADABS*; Bruker, 2000 ▶) *T*_min_ = 0.311, *T*_max_ = 1
8823 measured reflections
5772 independent reflections
5279 reflections with *I* > 2σ(*I*)
*R*_int_ = 0.023

#### Refinement

*R*[*F* ^2^ > 2σ(*F* ^2^)] = 0.031
*wR*(*F* ^2^) = 0.104
*S* = 1.07
5772 reflections
299 parameters
H-atom parameters constrained
Δρ_max_ = 1.62 e Å^−3^
Δρ_min_ = −1.26 e Å^−3^

### 

Data collection: *SMART* (Bruker, 2000 ▶); cell refinement: *SAINT* (Bruker, 2000 ▶); data reduction: *SAINT*; program(s) used to solve structure: *PATTY* in *DIRDIF92* (Beurskens *et al.*, 1992 ▶); program(s) used to refine structure: *SHELXL97* (Sheldrick, 2008 ▶); molecular graphics: *DIAMOND* (Brandenburg, 2006 ▶); software used to prepare material for publication: *publCIF* (Westrip, 2009 ▶).

## Supplementary Material

Crystal structure: contains datablocks global, I. DOI: 10.1107/S1600536809048466/sj2686sup1.cif

Structure factors: contains datablocks I. DOI: 10.1107/S1600536809048466/sj2686Isup2.hkl

Additional supplementary materials:  crystallographic information; 3D view; checkCIF report

## Figures and Tables

**Table:** 

Au—S1	2.3105 (11)
Au—P1	2.2509 (11)

**Table:** 

P1—Au—S1	177.00 (4)

**Fig. 1.**
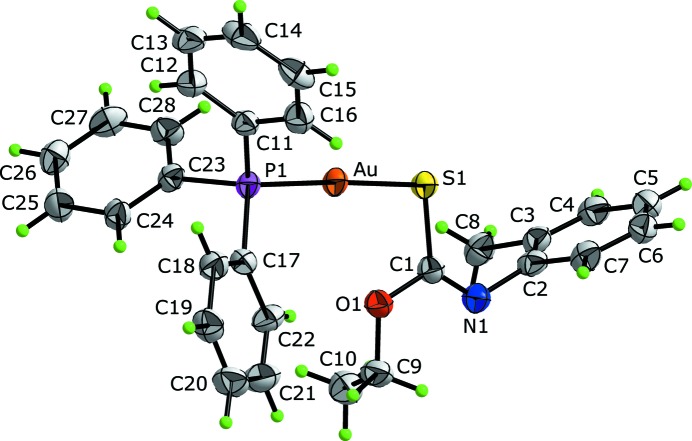
Molecular structure of (I) showing atom-labelling scheme and displacement ellipsoids at the 50% probability level.

**Fig. 2.**
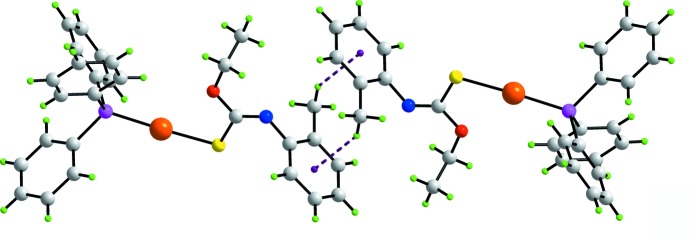
The supramolecular dimer in (I) mediated by C—H···π contacts (purple dashed lines). Colour code: Au, orange; S, yellow; P, pink; O, red; N, blue; C, grey; and H, green.

## supplementary crystallographic information

### Comment

The title compound, Ph_3_Au[SC(OEt)*N*(*o*-tolyl), (I), was synthesized during the course of systematic studies of phosphinegold(I) thiocarbamides (Ho *et al.* 2006; Ho & Tiekink, 2007; Kuan *et al.*, 2008)..

The gold atom in (I) exists in a linear geometry defined by an *S*,*P* donor set, Table 1 and Fig. 1. The small deviation from linearity is due to the close approach of the O1 atom to Au [Au···O = 2.936 (4) Å]. The anion, having C1—S1 = 1.768 (5) Å and C1N1 = 1.254 (6) Å, coordinates as a thiolate ligand. The configuration about the C1N1 double bond is *Z*.

In the crystal structure of (I), supramolecular dimers are formed between centrosymmetric pairs of *o*-tolyl residues owing to the presence of C—H···π interactions whereby the π system is defined by the (C2–C7) ring; C8–H8c···*Cg*^i^ = 2.67 Å, C8···*Cg*^i^ = 3.532 (6) Å with an angle at H8c = 148 ° for *i*: -1 - *x*, 1 - *y*, -*z*.

### Experimental

Compound (I) was prepared following the standard literature procedure from the reaction of Ph_3_AuCl and EtOC(*S*)*N*(*H*)(*o*-tolyl) in the presence of base (Hall *et al.*, 1993).

### Refinement

The H atoms were geometrically placed (C—H = 0.94–0.98 Å) and refined as riding with *U_iso_*(H) = 1.2–1.*5U_eq_*(C). The maximum and minimum residual electron density peaks of 1.62 and 1.26 e Å^-3^, respectively, were located 0.81 Å and 0.92 Å from the Au atom.

### Crystal data

**Table:**

[Au(C_10_H_12_NOS)(C_18_H_15_P)]	*Z* = 2
*M**_r_* = 653.50	*F*(000) = 640
Triclinic, *P*1	*D*_x_ = 1.716 Mg m^−^^3^
Hall symbol: -P 1	Mo *K*α radiation, λ = 0.71069 Å
*a* = 9.3378 (6) Å	Cell parameters from 5740 reflections
*b* = 10.1665 (6) Å	θ = 2.3–29.9°
*c* = 13.9711 (8) Å	µ = 5.98 mm^−^^1^
α = 95.514 (1)°	*T* = 223 K
β = 103.371 (1)°	Block, colourless
γ = 98.334 (1)°	0.24 × 0.13 × 0.03 mm
*V* = 1265.11 (13) Å^3^	

### Data collection

**Table:**

Bruker SMART CCD diffractometer	5772 independent reflections
Radiation source: fine-focus sealed tube	5279 reflections with *I* > 2σ(*I*)
graphite	*R*_int_ = 0.023
ω scans	θ_max_ = 27.5°, θ_min_ = 1.5°
Absorption correction: multi-scan (*SADABS*; Bruker, 2000)	*h* = −12→11
*T*_min_ = 0.311, *T*_max_ = 1	*k* = −10→13
8823 measured reflections	*l* = −17→18

### Refinement

**Table:**

Refinement on *F*^2^	Primary atom site location: structure-invariant direct methods
Least-squares matrix: full	Secondary atom site location: difference Fourier map
*R*[*F*^2^ > 2σ(*F*^2^)] = 0.031	Hydrogen site location: inferred from neighbouring sites
*wR*(*F*^2^) = 0.104	H-atom parameters constrained
*S* = 1.07	*w* = 1/[σ^2^(*F*_o_^2^) + (0.0692*P*)^2^] where *P* = (*F*_o_^2^ + 2*F*_c_^2^)/3
5772 reflections	(Δ/σ)_max_ = 0.001
299 parameters	Δρ_max_ = 1.62 e Å^−^^3^
0 restraints	Δρ_min_ = −1.26 e Å^−^^3^

### Special details

**Table:**

Geometry. All s.u.'s (except the s.u. in the dihedral angle between two l.s. planes) are estimated using the full covariance matrix. The cell s.u.'s are taken into account individually in the estimation of s.u.'s in distances, angles and torsion angles; correlations between s.u.'s in cell parameters are only used when they are defined by crystal symmetry. An approximate (isotropic) treatment of cell s.u.'s is used for estimating s.u.'s involving l.s. planes.
Refinement. Refinement of *F*^2^ against ALL reflections. The weighted *R*-factor *wR* and goodness of fit *S* are based on *F*^2^, conventional *R*-factors *R* are based on *F*, with *F* set to zero for negative *F*^2^. The threshold expression of *F*^2^ > 2σ(*F*^2^) is used only for calculating *R*-factors(gt) *etc*. and is not relevant to the choice of reflections for refinement. *R*-factors based on *F*^2^ are statistically about twice as large as those based on *F*, and *R*- factors based on ALL data will be even larger.

### Fractional atomic coordinates and isotropic or equivalent isotropic displacement parameters (Å^2^)

**Table:**

	*x*	*y*	*z*	*U*_iso_*/*U*_eq_	
Au	0.147930 (18)	0.358507 (16)	0.308910 (12)	0.03424 (8)	
S1	−0.00461 (14)	0.49405 (12)	0.22765 (9)	0.0352 (2)	
P1	0.28887 (13)	0.21714 (12)	0.38273 (9)	0.0305 (2)	
O1	−0.1318 (4)	0.2462 (3)	0.1600 (3)	0.0419 (8)	
N1	−0.2434 (5)	0.3967 (4)	0.0724 (3)	0.0392 (9)	
C1	−0.1424 (5)	0.3740 (5)	0.1420 (3)	0.0340 (9)	
C2	−0.2509 (5)	0.5300 (5)	0.0506 (4)	0.0368 (10)	
C3	−0.3452 (5)	0.6059 (5)	0.0885 (3)	0.0375 (10)	
C4	−0.3575 (6)	0.7297 (5)	0.0590 (4)	0.0435 (11)	
H4	−0.4198	0.7813	0.0840	0.052*	
C5	−0.2799 (6)	0.7804 (6)	−0.0070 (4)	0.0483 (13)	
H5	−0.2893	0.8658	−0.0256	0.058*	
C6	−0.1902 (6)	0.7060 (6)	−0.0448 (4)	0.0447 (12)	
H6	−0.1390	0.7395	−0.0903	0.054*	
C7	−0.1747 (5)	0.5813 (6)	−0.0160 (4)	0.0412 (11)	
H7	−0.1120	0.5307	−0.0417	0.049*	
C8	−0.4332 (6)	0.5472 (6)	0.1575 (4)	0.0461 (12)	
H8A	−0.4931	0.6107	0.1765	0.069*	
H8B	−0.3648	0.5289	0.2164	0.069*	
H8C	−0.4981	0.4645	0.1238	0.069*	
C9	−0.2431 (6)	0.1389 (5)	0.0974 (4)	0.0476 (12)	
H9A	−0.1996	0.0572	0.0923	0.057*	
H9B	−0.2735	0.1637	0.0305	0.057*	
C10	−0.3760 (7)	0.1122 (7)	0.1382 (5)	0.0568 (15)	
H10A	−0.4484	0.0400	0.0952	0.085*	
H10B	−0.4203	0.1925	0.1420	0.085*	
H10C	−0.3462	0.0866	0.2040	0.085*	
C11	0.4873 (5)	0.2811 (5)	0.4272 (4)	0.0336 (9)	
C12	0.5675 (6)	0.2763 (5)	0.5233 (4)	0.0425 (11)	
H12	0.5166	0.2442	0.5694	0.051*	
C13	0.7199 (6)	0.3175 (6)	0.5527 (4)	0.0490 (13)	
H13	0.7724	0.3139	0.6182	0.059*	
C14	0.7945 (6)	0.3639 (6)	0.4854 (5)	0.0523 (14)	
H14	0.8989	0.3902	0.5047	0.063*	
C15	0.7178 (6)	0.3723 (6)	0.3895 (5)	0.0509 (13)	
H15	0.7697	0.4056	0.3443	0.061*	
C16	0.5622 (6)	0.3310 (5)	0.3600 (4)	0.0449 (11)	
H16	0.5092	0.3372	0.2951	0.054*	
C17	0.2775 (5)	0.0655 (4)	0.2989 (3)	0.0317 (9)	
C18	0.3943 (6)	−0.0073 (5)	0.3093 (4)	0.0413 (11)	
H18	0.4832	0.0241	0.3587	0.050*	
C19	0.3800 (7)	−0.1259 (6)	0.2472 (4)	0.0479 (13)	
H19	0.4599	−0.1736	0.2534	0.058*	
C20	0.2469 (8)	−0.1739 (6)	0.1757 (5)	0.0550 (14)	
H20	0.2355	−0.2559	0.1351	0.066*	
C21	0.1319 (7)	−0.1011 (7)	0.1645 (5)	0.0578 (15)	
H21	0.0427	−0.1331	0.1155	0.069*	
C22	0.1467 (6)	0.0183 (6)	0.2245 (4)	0.0436 (11)	
H22	0.0685	0.0682	0.2153	0.052*	
C23	0.2305 (5)	0.1605 (5)	0.4895 (3)	0.0328 (9)	
C24	0.2240 (6)	0.0302 (5)	0.5088 (4)	0.0389 (10)	
H24	0.2465	−0.0348	0.4647	0.047*	
C25	0.1840 (6)	−0.0054 (6)	0.5941 (4)	0.0478 (12)	
H25	0.1777	−0.0949	0.6068	0.057*	
C26	0.1532 (6)	0.0910 (6)	0.6604 (4)	0.0467 (12)	
H26	0.1288	0.0670	0.7188	0.056*	
C27	0.1583 (7)	0.2195 (6)	0.6411 (4)	0.0502 (13)	
H27	0.1370	0.2843	0.6860	0.060*	
C28	0.1950 (6)	0.2562 (5)	0.5551 (4)	0.0425 (11)	
H28	0.1959	0.3449	0.5412	0.051*	

### Atomic displacement parameters (Å^2^)

**Table:**

	*U*^11^	*U*^22^	*U*^33^	*U*^12^	*U*^13^	*U*^23^
Au	0.03477 (12)	0.03251 (12)	0.03386 (12)	0.01184 (8)	0.00062 (8)	0.00697 (7)
S1	0.0354 (6)	0.0300 (5)	0.0369 (6)	0.0114 (4)	−0.0012 (5)	0.0052 (4)
P1	0.0305 (5)	0.0294 (6)	0.0300 (6)	0.0095 (4)	0.0012 (4)	0.0054 (4)
O1	0.0446 (19)	0.0308 (17)	0.0437 (19)	0.0065 (14)	−0.0023 (15)	0.0060 (14)
N1	0.038 (2)	0.037 (2)	0.041 (2)	0.0108 (17)	0.0020 (17)	0.0072 (17)
C1	0.035 (2)	0.030 (2)	0.036 (2)	0.0089 (18)	0.0053 (19)	0.0039 (18)
C2	0.028 (2)	0.040 (3)	0.036 (2)	0.0070 (19)	−0.0033 (18)	0.0013 (19)
C3	0.039 (2)	0.041 (3)	0.030 (2)	0.011 (2)	0.0010 (19)	0.0030 (19)
C4	0.044 (3)	0.044 (3)	0.040 (3)	0.012 (2)	0.005 (2)	−0.001 (2)
C5	0.052 (3)	0.038 (3)	0.047 (3)	0.003 (2)	0.000 (2)	0.008 (2)
C6	0.044 (3)	0.045 (3)	0.043 (3)	0.004 (2)	0.004 (2)	0.012 (2)
C7	0.034 (2)	0.047 (3)	0.043 (3)	0.016 (2)	0.004 (2)	0.007 (2)
C8	0.048 (3)	0.053 (3)	0.039 (3)	0.015 (2)	0.011 (2)	0.005 (2)
C9	0.053 (3)	0.031 (2)	0.050 (3)	0.007 (2)	−0.002 (2)	−0.001 (2)
C10	0.047 (3)	0.053 (3)	0.065 (4)	0.005 (3)	0.002 (3)	0.013 (3)
C11	0.030 (2)	0.029 (2)	0.038 (2)	0.0040 (17)	0.0017 (18)	0.0047 (18)
C12	0.042 (3)	0.038 (3)	0.041 (3)	0.000 (2)	0.002 (2)	0.005 (2)
C13	0.039 (3)	0.042 (3)	0.052 (3)	0.005 (2)	−0.013 (2)	0.001 (2)
C14	0.034 (3)	0.038 (3)	0.077 (4)	0.000 (2)	0.002 (3)	0.004 (3)
C15	0.046 (3)	0.040 (3)	0.068 (4)	−0.002 (2)	0.023 (3)	0.007 (3)
C16	0.047 (3)	0.041 (3)	0.047 (3)	0.010 (2)	0.010 (2)	0.010 (2)
C17	0.033 (2)	0.028 (2)	0.034 (2)	0.0051 (17)	0.0085 (18)	0.0054 (17)
C18	0.046 (3)	0.041 (3)	0.036 (2)	0.009 (2)	0.008 (2)	0.003 (2)
C19	0.063 (4)	0.039 (3)	0.049 (3)	0.016 (3)	0.023 (3)	0.007 (2)
C20	0.075 (4)	0.039 (3)	0.051 (3)	0.005 (3)	0.023 (3)	−0.006 (2)
C21	0.057 (3)	0.058 (4)	0.047 (3)	−0.002 (3)	0.002 (3)	−0.007 (3)
C22	0.036 (2)	0.049 (3)	0.037 (3)	0.001 (2)	0.000 (2)	−0.001 (2)
C23	0.032 (2)	0.036 (2)	0.031 (2)	0.0124 (18)	0.0045 (17)	0.0039 (18)
C24	0.044 (3)	0.037 (2)	0.039 (3)	0.014 (2)	0.009 (2)	0.010 (2)
C25	0.056 (3)	0.045 (3)	0.046 (3)	0.010 (2)	0.015 (2)	0.013 (2)
C26	0.044 (3)	0.058 (3)	0.041 (3)	0.010 (2)	0.014 (2)	0.010 (2)
C27	0.050 (3)	0.059 (4)	0.042 (3)	0.014 (3)	0.016 (2)	−0.007 (3)
C28	0.047 (3)	0.037 (3)	0.045 (3)	0.015 (2)	0.011 (2)	−0.001 (2)

### Geometric parameters (Å, °)

**Table:**

Au—S1	2.3105 (11)	C12—C13	1.375 (7)
Au—P1	2.2509 (11)	C12—H12	0.9400
S1—C1	1.768 (5)	C13—C14	1.373 (9)
P1—C11	1.812 (5)	C13—H13	0.9400
P1—C23	1.817 (5)	C14—C15	1.382 (9)
P1—C17	1.819 (5)	C14—H14	0.9400
O1—C1	1.360 (6)	C15—C16	1.403 (8)
O1—C9	1.450 (6)	C15—H15	0.9400
N1—C1	1.254 (6)	C16—H16	0.9400
N1—C2	1.425 (6)	C17—C18	1.392 (7)
C2—C7	1.391 (7)	C17—C22	1.396 (6)
C2—C3	1.410 (7)	C18—C19	1.386 (8)
C3—C4	1.374 (7)	C18—H18	0.9400
C3—C8	1.514 (7)	C19—C20	1.392 (9)
C4—C5	1.390 (8)	C19—H19	0.9400
C4—H4	0.9400	C20—C21	1.379 (9)
C5—C6	1.366 (8)	C20—H20	0.9400
C5—H5	0.9400	C21—C22	1.377 (8)
C6—C7	1.384 (7)	C21—H21	0.9400
C6—H6	0.9400	C22—H22	0.9400
C7—H7	0.9400	C23—C24	1.372 (7)
C8—H8A	0.9700	C23—C28	1.401 (7)
C8—H8B	0.9700	C24—C25	1.393 (7)
C8—H8C	0.9700	C24—H24	0.9400
C9—C10	1.482 (9)	C25—C26	1.390 (8)
C9—H9A	0.9800	C25—H25	0.9400
C9—H9B	0.9800	C26—C27	1.356 (9)
C10—H10A	0.9700	C26—H26	0.9400
C10—H10B	0.9700	C27—C28	1.392 (8)
C10—H10C	0.9700	C27—H27	0.9400
C11—C16	1.387 (7)	C28—H28	0.9400
C11—C12	1.387 (7)		
			
P1—Au—S1	177.00 (4)	C13—C12—C11	121.4 (5)
C1—S1—Au	101.39 (16)	C13—C12—H12	119.3
C11—P1—C23	105.3 (2)	C11—C12—H12	119.3
C11—P1—C17	104.4 (2)	C14—C13—C12	119.4 (5)
C23—P1—C17	105.6 (2)	C14—C13—H13	120.3
C11—P1—Au	116.15 (15)	C12—C13—H13	120.3
C23—P1—Au	112.80 (15)	C13—C14—C15	120.7 (5)
C17—P1—Au	111.68 (15)	C13—C14—H14	119.6
C1—O1—C9	117.5 (4)	C15—C14—H14	119.6
C1—N1—C2	120.8 (4)	C14—C15—C16	119.7 (5)
N1—C1—O1	120.6 (4)	C14—C15—H15	120.1
N1—C1—S1	127.0 (4)	C16—C15—H15	120.1
O1—C1—S1	112.4 (3)	C11—C16—C15	119.5 (5)
C7—C2—C3	119.5 (5)	C11—C16—H16	120.2
C7—C2—N1	119.5 (5)	C15—C16—H16	120.2
C3—C2—N1	120.7 (5)	C18—C17—C22	119.2 (5)
C4—C3—C2	118.4 (5)	C18—C17—P1	121.5 (4)
C4—C3—C8	122.2 (5)	C22—C17—P1	119.3 (4)
C2—C3—C8	119.3 (5)	C19—C18—C17	120.4 (5)
C3—C4—C5	121.6 (5)	C19—C18—H18	119.8
C3—C4—H4	119.2	C17—C18—H18	119.8
C5—C4—H4	119.2	C18—C19—C20	119.8 (6)
C6—C5—C4	119.9 (5)	C18—C19—H19	120.1
C6—C5—H5	120.1	C20—C19—H19	120.1
C4—C5—H5	120.1	C21—C20—C19	119.9 (5)
C5—C6—C7	120.0 (5)	C21—C20—H20	120.0
C5—C6—H6	120.0	C19—C20—H20	120.0
C7—C6—H6	120.0	C22—C21—C20	120.5 (6)
C6—C7—C2	120.6 (5)	C22—C21—H21	119.7
C6—C7—H7	119.7	C20—C21—H21	119.7
C2—C7—H7	119.7	C21—C22—C17	120.2 (5)
C3—C8—H8A	109.5	C21—C22—H22	119.9
C3—C8—H8B	109.5	C17—C22—H22	119.9
H8A—C8—H8B	109.5	C24—C23—C28	119.6 (5)
C3—C8—H8C	109.5	C24—C23—P1	122.8 (4)
H8A—C8—H8C	109.5	C28—C23—P1	117.6 (4)
H8B—C8—H8C	109.5	C23—C24—C25	119.9 (5)
O1—C9—C10	111.1 (5)	C23—C24—H24	120.1
O1—C9—H9A	109.4	C25—C24—H24	120.1
C10—C9—H9A	109.4	C26—C25—C24	120.2 (5)
O1—C9—H9B	109.4	C26—C25—H25	119.9
C10—C9—H9B	109.4	C24—C25—H25	119.9
H9A—C9—H9B	108.0	C27—C26—C25	120.1 (5)
C9—C10—H10A	109.5	C27—C26—H26	119.9
C9—C10—H10B	109.5	C25—C26—H26	119.9
H10A—C10—H10B	109.5	C26—C27—C28	120.4 (5)
C9—C10—H10C	109.5	C26—C27—H27	119.8
H10A—C10—H10C	109.5	C28—C27—H27	119.8
H10B—C10—H10C	109.5	C27—C28—C23	119.8 (5)
C16—C11—C12	119.2 (5)	C27—C28—H28	120.1
C16—C11—P1	117.8 (4)	C23—C28—H28	120.1
C12—C11—P1	123.0 (4)		
			
C2—N1—C1—O1	−177.2 (4)	C12—C11—C16—C15	1.7 (8)
C2—N1—C1—S1	4.3 (7)	P1—C11—C16—C15	−175.3 (4)
C9—O1—C1—N1	−1.4 (7)	C14—C15—C16—C11	−0.5 (8)
C9—O1—C1—S1	177.3 (4)	C11—P1—C17—C18	26.2 (4)
Au—S1—C1—N1	−171.6 (4)	C23—P1—C17—C18	−84.6 (4)
Au—S1—C1—O1	9.8 (4)	Au—P1—C17—C18	152.4 (4)
C1—N1—C2—C7	89.5 (6)	C11—P1—C17—C22	−156.2 (4)
C1—N1—C2—C3	−96.3 (6)	C23—P1—C17—C22	93.0 (4)
C7—C2—C3—C4	−0.7 (7)	Au—P1—C17—C22	−29.9 (4)
N1—C2—C3—C4	−174.9 (4)	C22—C17—C18—C19	−0.7 (7)
C7—C2—C3—C8	177.4 (5)	P1—C17—C18—C19	177.0 (4)
N1—C2—C3—C8	3.1 (7)	C17—C18—C19—C20	−1.5 (8)
C2—C3—C4—C5	0.2 (8)	C18—C19—C20—C21	2.3 (9)
C8—C3—C4—C5	−177.8 (5)	C19—C20—C21—C22	−0.9 (10)
C3—C4—C5—C6	0.7 (8)	C20—C21—C22—C17	−1.3 (9)
C4—C5—C6—C7	−1.1 (8)	C18—C17—C22—C21	2.1 (8)
C5—C6—C7—C2	0.6 (8)	P1—C17—C22—C21	−175.6 (4)
C3—C2—C7—C6	0.3 (7)	C11—P1—C23—C24	−93.8 (4)
N1—C2—C7—C6	174.6 (5)	C17—P1—C23—C24	16.3 (5)
C1—O1—C9—C10	−88.1 (6)	Au—P1—C23—C24	138.5 (4)
C23—P1—C11—C16	179.7 (4)	C11—P1—C23—C28	84.5 (4)
C17—P1—C11—C16	68.7 (4)	C17—P1—C23—C28	−165.4 (4)
Au—P1—C11—C16	−54.7 (4)	Au—P1—C23—C28	−43.2 (4)
C23—P1—C11—C12	2.8 (5)	C28—C23—C24—C25	−0.8 (8)
C17—P1—C11—C12	−108.2 (4)	P1—C23—C24—C25	177.5 (4)
Au—P1—C11—C12	128.4 (4)	C23—C24—C25—C26	−1.1 (8)
C16—C11—C12—C13	−1.3 (8)	C24—C25—C26—C27	1.7 (9)
P1—C11—C12—C13	175.5 (4)	C25—C26—C27—C28	−0.3 (9)
C11—C12—C13—C14	−0.3 (9)	C26—C27—C28—C23	−1.6 (9)
C12—C13—C14—C15	1.4 (9)	C24—C23—C28—C27	2.2 (8)
C13—C14—C15—C16	−1.0 (9)	P1—C23—C28—C27	−176.2 (4)

## References

[bb1] Beurskens, P. T., Admiraal, G., Beurskens, G., Bosman, W. P., Garcia-Granda, S., Gould, R. O., Smits, J. M. M. & Smykalla, C. (1992). *The *DIRDIF* Program System*. Technical Report. Crystallography Laboratory, University of Nijmegen, The Netherlands.

[bb2] Brandenburg, K. (2006). *DIAMOND*. Crystal Impact GbR, Bonn, Germany.

[bb3] Bruker (2000). *SMART*, *SAINT* and *SADABS*. Bruker AXS Inc., Madison, Wisconsin, U. S. A.

[bb4] Hall, V. J., Siasios, G. & Tiekink, E. R. T. (1993). *Aust. J. Chem.* **46**, 561–570.

[bb5] Ho, S. Y., Cheng, E. C.-C., Tiekink, E. R. T. & Yam, V. W.-W. (2006). *Inorg. Chem.* **45**, 8165–8174. 10.1021/ic0608243 16999414

[bb6] Ho, S. Y. & Tiekink, E. R. T. (2007). *CrystEngComm*, **9**, 368–378.

[bb7] Kuan, F. S., Ho, S. Y., Tadbuppa, P. P. & Tiekink, E. R. T. (2008). *CrystEngComm*, **10**, 548–564.

[bb8] Sheldrick, G. M. (2008). *Acta Cryst.* A**64**, 112–122. 10.1107/S0108767307043930 18156677

[bb9] Westrip, S. P. (2009). *publCIF*. In preparation.

